# In-situ simulations to detect patient safety threats during in-hospital cardiac arrest

**DOI:** 10.1016/j.resplu.2023.100410

**Published:** 2023-06-08

**Authors:** Mathilde Stærk, Kasper G. Lauridsen, Josephine Johnsen, Bo Løfgren, Kristian Krogh

**Affiliations:** a Department of Emergency Medicine, Gødstrup Hospital, Denmark; b Department of Medicine, Randers Regional Hospital, Denmark; c Education and Research, Randers Regional Hospital, Denmark; d Research Center for Emergency Medicine, Aarhus University Hospital, Denmark; e Department of Anesthesiology and Critical Care Medicine, Children’s Hospital of Philadelphia, USA; f Department of Internal Medicine, Diagnostic Centre, Silkeborg Regional Hospital, Denmark; g Department of Clinical Medicine, Aarhus University, Denmark; h Department of Anaesthesiology and Intensive Care, Aarhus University Hospital, Denmark

## Abstract

**Introduction:**

Errors during treatment may affect patient outcomes and can include errors in treatment algorithms, teamwork, and system errors. In-hospital cardiac arrests (IHCA) require immediate and effective treatment, and delays are known to reduce survival. In-situ simulation is a tool that can be used to study emergency responses, including IHCA. We investigated system errors discovered during unannounced in-situ simulated IHCA.

**Method:**

This multicenter cohort study included unannounced, full-scale IHCA in-situ simulations followed by a debriefing based on PEARLS with plus-delta used in the analysis phase. Simulations and debriefings were video-recorded for subsequent analysis. System errors observed were categorized by thematic analysis and analyzed for clinical implications. Errors related to treatment algorithm and clinical performance were excluded.

**Results:**

We conducted 36 in-situ simulations across 4 hospitals with a total discovery of 30 system errors. On average, we discovered 0.8 system errors per simulation within the categories: human, organizational, hardware, or software errors. Of these, 25 errors (83%) had direct treatment consequences. System errors caused treatment delays in 15 cases, a need for alternative actions in 6 cases, omission of actions in 4 cases, and other consequences in 5 cases.

**Conclusion:**

Using unannounced in-situ simulations, we identified almost one system error per simulation, and most of these errors were deemed to impact treatment negatively. The errors affected treatment by either causing delays, need for alternative treatment options, or omitting treatment actions. We suggest that hospitals focus on the need for regular testing of the emergency response by conducting full-scale unannounced in-situ simulations. This should be a priority to improve patient safety and care.

## Introduction

Handling a medical emergency is a stressful situation with a risk of errors. In the USA, it is estimated that nearly 100.000 people/year die due to medical errors, where many could have been prevented.^1^ Errors frequently occur during in-hospital cardiac arrest (IHCA), and are associated with worse survival outcomes.^2^ IHCA calls for effective and immediate treatment, and delays lower the chance of survival.^3^, ^4^, ^5^

Errors can relate to many aspects of resuscitation, such as the treatment algorithm, communication, teamwork, and the overall system. While errors related to the treatment algorithm, communication, and teamwork can be limited by continuous education and training of the staff, system errors require solutions on an organizational level. Today, most research on errors during resuscitation focuses on human errors affecting treatment algorithms and teamwork.^6^, ^7^, ^8^ However, a well-functioning organization with a minimum of system errors is also needed.

In-situ simulations can serve as a tool to investigate emergency responses, improve workflow, and test the organization.^9^, ^10^, ^11^, ^12^ Thus, in-situ simulations provide the possibility to adjust the procedures and rectify errors discovered during in-situ simulations by identifying hazardous and latent safety threats to avoid errors from happening in real situations.^11^, ^13^, ^14^, ^15^, ^16^, ^17^ In this study, we investigate system errors discovered during unannounced in-situ simulated IHCA conducted in four Danish hospitals.

## Methods

### Study design

This is a post-hoc analysis of a prospective, multicenter simulation-based study consisting of full-scale unannounced in-situ simulated IHCA conducted at four hospitals.^18^ The method is described in detail elsewhere.^18^, ^19^

### Setting

We conducted full-scale in-situ simulations at one university hospital and three regional hospitals in departments (in-patient and out-patient clinical areas) with an automated external defibrillator (AED) during workdays, weekends, day shifts, and night shifts. We excluded intensive care units, cardiology, pediatric, and psychiatric departments. The nurse manager on the specific ward assigned a patient room for the simulation on the day of simulation. The location, time, and content of the simulation were unknown to all other staff. During the study period, all hospital staff that could be involved in the in-situ simulations were provided with written information about the possible occurrence of an in-situ simulation and instructed to act as in real life in case of a simulation.

A full-body manikin (Resusci Anne QCPR AED with Airway Head, Laerdal Medical, Stavanger, Norway) was placed in a hospital bed, and a normal patient call was activated in the room. A nurse/nurse assistant responded and was briefed with a short patient history. The nurse/nurse assistant was instructed to assess the patient, that presented as unresponsive and without breathing. The simulation consisted of a shockable scenario and continued until the third rhythm analysis by the cardiac arrest team where return of spontaneous circulation was obtained. It was possible to conduct actions as in real life, e.g., activate the hospital’s cardiac arrest team, retrieve information from the patient record (including e.g., blood samples, an electrocardiogram, chest x-ray), administer intravenous medication, perform intubation, and use the hospital defibrillators. Participants needed to retrieve and use their normal equipment and did not receive any feedback nor help during the simulations. All simulations were followed by a debriefing based on PEARLS with plus-delta included in all debriefings. Debriefers were experienced and certified simulation instructors and did not probe for systems errors unless brought up by participating staff members.^20^, ^21^

### Data collection

We video-recorded the in-situ simulations and debriefings using two cameras capable of capturing 180-degree video (GoPro Hero 5 Black, San Mateo, CA, USA). An additional camera was used during the debriefings. The videos were reviewed by two independent researchers following a predetermined template with an included note field for other observations including system errors. Videos were re-reviewed if any disagreement. These data were the source of the latent system errors along with the debriefing transcripts. We conducted a thematic analysis of the errors to identify overall themes. Errors with an urgent risk to patient safety were reported to the department management within 24 hours of the simulation to be rectified immediately.

### Ethics

The study was approved by the Danish Data Protection Agency (j.no. 1-16-02-367-18) and deemed exempt from ethical committee approval and individual consent (j.no. 141/2017). Hospital administrations gave permission, and hospital departments were informed about the study. Safety precautions were taken to secure patient safety during simulations.^18^, ^19^

## Results

We conducted 36 in-situ simulations across four different hospitals evenly distributed on wards that had an AED with an average of 4 simulations per department July 2018 to December 2020. In total, 30 in-situ simulations were complete, whereas 6 in-situ simulations were interrupted due to emergency calls/acute patients. We identified 30 system errors, equal to 0.8 system error per simulation, related to the categories: human errors (*n* = 6, 20%), organizational errors (*n* = 3, 10%), hardware errors (*n* = 5, 17%), and software errors (*n* = 16%, 53%) (Table 1). Overall, 15 (50%) errors resulted in delayed actions, e.g., the AED did not have defibrillation electrodes attached, causing delayed defibrillation, 6 (20%) errors resulted in an alternative action conducted, e.g., the emergency alert system did not work, and the staff had to leave the patient room to alert colleagues, 4 (13%) errors resulted in actions having to be omitted, e.g., the laryngoscope could not be located, and intubation was therefore not performed, and 5 (17%) errors had other consequences. All errors, but one (wrongful rhythm analysis by AED), were preventable at a hospital level. Errors related to the treatment algorithm and clinical performance are reported elsewhere.^18^, ^19^

**Table 1:** Errors discovered during in-situ simulations and how they affected treatment. Foot note: *‘Code call’: a notification of a cardiac arrest and location to the cardiac arrest team members. ‘Emergency alerting call’: a prioritized telephone call to all nursing telephones in the department, making it possible to talk to all nursing staff at once. ‘Local emergency alert system’: panels in the patient room with the possibility to send a notification of an emergency/acute situation to all nursing staff at the specific department*.

**Error category**	**Consequence**
**Human errors**	
Step stool for providing chest compressions was misplaced and could not be found	Alternative action
‘Code call’ for the cardiac arrest team was sent with the wrong location and the cardiac arrest team therefore arrived at wrongful site	Delayed action
Anesthetist equipment was not fully stocked despite equipment being sealed	Action omitted
Pressure bags were missing in the resuscitation trolley	Alternative action
Emergency medicine box was expired	Other
The AED did not have defibrillation electrodes	Delayed action
**Organizational errors**	
The laryngoscope could not be located in the resuscitation trolley due to the resuscitation trolley being unorganized and filled with too many items	Action omitted
Potassium was not available neither in the emergency equipment nor in the department	Action omitted
Staff did not have telephones, nor was a telephone available in the patient room, which delayed the process of calling the cardiac arrest team.	Delayed action
**Hardware errors**	
Therapy cable on defibrillator had a loose connection	Delayed action
Therapy cable on defibrillator had a loose connection	Delayed action
Defibrillator batteries were old and could not provide sufficient power for defibrillation. Defibrillator needed connection to a power outlet to defibrillate	Delayed action
The mechanical chest compression device’s claw locks were jammed, and the device, therefore, was difficult to attach	Delayed action
AED was in full-automatic mode instead of semi-automatic mode	Other
**Software errors**	
The ‘emergency alerting call’ did not overrule regular phone calls	Delayed action
The ‘emergency alerting call’ was not received on all telephones	Delayed action
A telephone could not accept a received ‘emergency alerting call’	Delayed action
The local emergency alert was not received on all pagers/telephones	Delayed action
The switchboard operator system did not register that the internal medicine specialist had accepted the cardiac arrest code call despite having accepted this on the telephone system	Other
Code call was not received by the orderly and the anesthetist	Delayed action
The anesthetist could not accept the code call on the telephone	Other
The anesthetist, nurse anesthetist, and orderly could not report ‘arrived’ on the telephone but only accept the code call	Other
The local emergency alert system at the specific bed site did not work	Alternative action
The local emergency alert system at the specific bed site did not work	Alternative action
The local emergency alert system in the patient room did not work	Alternative action
The local emergency alert system in the patient room did not work	Alternative action
Prioritization system for elevators in case of emergencies did not function. The elevator instead stayed stock at one floor	Delayed action
Prioritization system for elevators in case of emergencies did not function. The elevator went down despite being directed upwards	Delayed action
Prioritization system for elevators did not function. This delayed both the orderly, the anesthetist, and nurse anesthetist	Delayed action
AED recommended ‘no shock’ despite analyzing on a shockable rhythm (ventricular fibrillation)	Action omitted

## Discussion

During in-situ simulations, we found errors causing delays in treatment, need for alternative actions, omitted actions, or other consequences. Most system errors influenced the treatment and could potentially have affected clinical patient outcomes. On average, we identified one system error per in-situ simulation. This is less than previous studies that have identified up to 2.1 latent safety threats per simulation.^11^, ^12^, ^22^ However, these studies also report errors related to the treatment algorithm and teamwork. We have reported errors related to the initial treatment algorithm, and defibrillation elsewhere.^18^, ^19^ If all errors were reported together, our study would also have identified more errors per simulation. Importantly, the system errors we identified are similar to those reported in other studies such as cardiac arrest calling procedures, equipment malfunctions, or delays in locating the equipment during resuscitation.^23^, ^24^ Unannounced in-situ simulations may therefore be an important method to identify errors that may threaten patient safety.

The system errors mainly resulted in delays of treatment. This may be critical for the survival of the patient, as survival depends on many time-sensitive factors, e.g., time to first chest compression, defibrillation, cardiac arrest team arrival, adrenaline administration, etc.^3^, ^5^, ^25^, ^26^, ^27^, ^28^ Many of the errors we discovered related to the process of alerting relevant staff. Some of these errors resulted in the delayed arrival of relevant staff, whilst others caused the need for staff at site to leave the patient room to retrieve help as the alarm system in the room could not be activated. This resulted in delayed initiation of basic life support and increased chest compression pauses which negatively affects patient outcomes.^3^, ^5^, ^29^

The system errors could almost all be rectified at a hospital level and therefore may mitigate potential patient harm in case of a real IHCA. For example, some of the system errors relating to equipment being misplaced or difficult to find may be solved by providing knowledge about where the equipment is located, and the importance of the equipment being correctly replaced after use. Other system errors related to software issues could be solved by adjusting the software or implementing other software solutions, e.g., one hospital struggled with the alarm system on the telephones, which other hospitals did not experience as they used a newer software solution for telephone alerts.

The process of identifying errors is a challenging task, which may often be divided into smaller “parts”, e.g., only testing the telephone alerting system and not the alerting system in the patient rooms. Current training in the emergency response is most often conducted in a simulation center, thereby excluding the possibility of discovering system errors by only focusing on finding and adjusting treatment errors, teamwork, communication, etc. The system errors discovered in our study would not have been identified if the simulations were conducted at a simulation center, as many system errors related to the alerting process and staff transportation to the location. In-situ simulation can be used as a practical and important tool to test the organizational response, either in full or partwise and can be used to study and train the treatment algorithm and knowledge and skills among responders. Especially, unannounced in-situ simulations can mimic the clinical setting to an extent that provides the ultimate “pressure-test” of the system. Previously, it has been reported how in-situ simulations have contributed to the discovery of errors, improvement of processes, and general training of staff’s knowledge and skills.^11^, ^12^, ^13^, ^14^, ^22^, ^30^

### Limitations

This study was not designed to look for system errors or latent safety threats specifically but rather to document resuscitation from the initial patient contact in a standardized, unannounced shockable cardiac arrest scenario. Despite the participant-focused approach using plus-delta debriefing, we identified many system errors and safety issues. This highlights the potential for identifying latent safety threats using in-situ simulations that ideally should be preceded by an assessment of the clinical workspace and with a scenario designed to highlight potential risks or issues.

## Conclusion

Using unannounced in-situ simulations, we identified almost one system error per simulation, and most of these errors were deemed to impact treatment negatively. The errors affected treatment by either causing delays, need for alternative treatment options, or omitting treatment actions. We suggest that hospitals utilize full-scale unannounced in-situ simulations to minimize system errors and improve patient safety.

## CRediT authorship contribution statement

**Mathilde Stærk:** Conceptualization, Methodology, Resources, Investigation, Writing – original draft, Writing – review & editing, Project administration. **Kasper G. Lauridsen:** Conceptualization, Methodology, Investigation, Writing – review & editing, Supervision. **Josephine Johnsen:** Investigation, Writing – review & editing. **Bo Løfgren:** Conceptualization, Methodology, Resources, Writing – review & editing, Supervision. **Kristian Krogh:** Conceptualization, Methodology, Investigation, Writing – review & editing, Supervision.

## Declaration of Competing Interest

The authors declare that they have no known competing financial interests or personal relationships that could have appeared to influence the work reported in this paper.
